# Celiac Disease Genomic, Environmental, Microbiome, and Metabolomic (CDGEMM) Study Design: Approach to the Future of Personalized Prevention of Celiac Disease

**DOI:** 10.3390/nu7115470

**Published:** 2015-11-11

**Authors:** Maureen M. Leonard, Stephanie Camhi, Tania B. Huedo-Medina, Alessio Fasano

**Affiliations:** 1 Center for Celiac Research, Massachusetts General Hospital for Children, Boston, MA 02114, USA; sscamhi@partners.org (S.C.); afasano@mgh.harvard.edu (A.F.); 2 Mucosal Immunology and Biology Research Center, Massachusetts General Hospital and Division of Pediatric Gastroenterology and Nutrition, Massachusetts General Hospital for Children, Boston, MA 02114, USA; 3 Allied Health Sciences Department, University of Connecticut, Storrs, CT 06269, USA; tania.huedo-medina@uconn.edu

**Keywords:** celiac, gluten, microbiome, metabolomic, environmental, genetic, personalized medicine, prospective, cohort, design

## Abstract

In the past it was believed that genetic predisposition and exposure to gluten were necessary and sufficient to develop celiac disease (CD). Recent studies however suggest that loss of gluten tolerance can occur at any time in life as a consequence of other environmental stimuli. Many environmental factors known to influence the composition of the intestinal microbiota are also suggested to play a role in the development of CD. These include birthing delivery mode, infant feeding, and antibiotic use. To date no large-scale longitudinal studies have defined if and how gut microbiota composition and metabolomic profiles may influence the loss of gluten tolerance and subsequent onset of CD in genetically-susceptible individuals. Here we describe a prospective, multicenter, longitudinal study of infants at risk for CD which will employ a blend of basic and applied studies to yield fundamental insights into the role of the gut microbiome as an additional factor that may play a key role in early steps involved in the onset of autoimmune disease.

## 1. Introduction

Celiac disease (CD) is an autoimmune enteropathy triggered by the ingestion of gluten containing grains (*i.e.*, wheat, barley, and rye) in genetically susceptible individuals [1]. CD represents a unique model of autoimmune disease as, in contrast to other autoimmune diseases, the triggering environmental factor (gluten), a close genetic association with human leukocyte antigen (HLA) genes (DQ2 or DQ8), and a highly specific humoral autoimmune response (autoantibodies to tissue transglutaminase) are known [1]. However, the early steps following intestinal mucosal exposure to gluten leading to the loss of tolerance and the development of the autoimmune process are still largely unknown. Studies now suggest that this loss of gluten tolerance does not always occur at the time of gluten introduction in the diet of genetically susceptible individuals; rather it can occur at any time in life as a consequence of other unknown environmental stimuli [2].

A proof of concept study, published by our group, has shown that a unique interplay between a peculiar microbiota and host may lead to alterations in metabolic pathways that result in the production of specific metabolites prior to the onset of autoimmune disease [3]. The intestinal microbiome is essential to the development of the immune system and begins to assemble in utero. As early as the first year after birth, the microbiota has begun to develop into an adult-like pattern, suggesting that environmental influences in infancy may have important lasting effects [4]. Many environmental factors known to influence the composition of the intestinal microbiota are also thought to play a role in the development of CD. These include birthing delivery mode, infant feeding type, history of infection, and antibiotic use [5,6,7,8,9,10,11].

The composition of the microbiota in infants at risk of developing autoimmune disease is altered when compared to infants without the genetic risk [3,12]. A previous study found that compared to control infants with a non-selected genetic background, at-risk subjects had a decreased representation of Bacteriodetes and a higher abundance of Firmicutes [3]. Their microbiota showed a delay in maturation at two years of age [3] while the maturation was complete in not at-risk infants at one year [4]. Additionally, this same study showed that infants who developed autoimmunity had decreased lactate signals in their stools coincident with a diminished representation in Lactobacillus species in their microbiome and that preceded the first detection of positive antibodies [3]. Early microbiota alterations in genetically-predisposed infants were also suggested in a recent study that compared microbial communities in infants carrying the DQ2 haplotype with infants who did not carry a compatible haplotype. Distinct differences between the microbiota composition at one month of age were observed, with infants who carry DQ2 showing a higher abundance of Firmicutes and Proteobacteria compared to infants without a genetic predisposition [12].

Two recent landmark studies which prospectively screened infants, with a first degree family member with CD, from birth found that CD develops quite early in life in this risk group, further supporting the notion that early environmental factors may be very important in the development of CD. These studies found that 16% of infants who have a first degree relative with CD and who carry HLA DQ2 and/or DQ8 will develop CD by age five, most of whom will be diagnosed by age three [5,6]. These studies further demonstrated that 38% of infants who are first-degree relatives of CD patients and who carry two copies of DQ2 will develop CD by age five [5,6].

The major limitation in performing pre-clinical studies pertinent to human gastrointestinal inflammatory diseases is the recent appreciation that animal models do not completely recapitulate the complexity of host-microbiota interactions that influence activation of specific metabolic pathways dictating the tolerance to immune response balance in humans. To date, no large-scale, longitudinal studies have defined if and how gut microbiota composition and metabolomic profiles may influence the loss of gluten tolerance and subsequent onset of CD in genetically susceptible subjects. Therefore, we propose to investigate the role of the developing intestinal microbiome and the resulting metabolome as additional factors that may play a key role in the onset of and predisposition to CD autoimmunity.

### 1.1. Objective

The objective of Celiac Disease Genomic, Environmental, Microbiome, and Metabolomic (CDGEMM) is to understand the role of the gut microbiome as an additional factor that may play a key role in early steps involved in the development of autoimmune disease. This prospective, longitudinal, observational study will serve as a basis for investigating the natural history of CD and other autoimmune disorders. We hypothesize that the combination of introduction of gluten into the diet and particular microbiota composition of infants genetically at risk for CD activates specific metabolic pathways that can contribute to the loss of gluten tolerance and to the onset of autoimmunity, as reflected by specific metabolomic phenotypes. Our ultimate goal is to identify and validate specific microbiome and metabolomic profiles that can predict loss of tolerance in children genetically at risk of autoimmunity. This study will serve to provide the foundation of knowledge necessary in order to one day implement early preventive interventions to re-establish tolerance and prevent CD.

### 1.2. Specific Aims

The three specific aims of CDGEMM are:

1. To study modifications of the infants’ microbiome in relation to specific environmental factors, presence or absence of HLA DQ2 and/or DQ8 predisposing genes, and in relation to tolerance *vs.* immune response leading to the autoimmune intestinal insult typical of CD;
2. To study the infants’ metabolomic phenotype variation in relation to tolerance *vs.* immune response leading to the autoimmune intestinal insult typical of CD; and
3. To investigate the impact of specific bacteria-derived metabolites on gut mucosal molecular pathways leading to the early steps of CD pathogenesis.

## 2. Study Design

**Figure 1:**
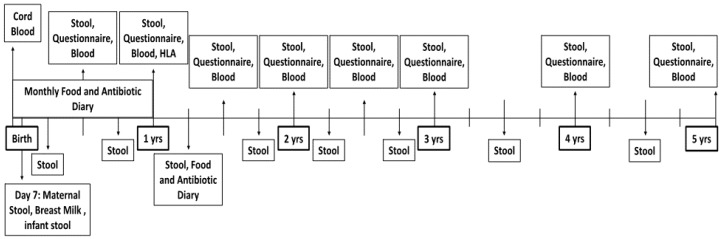
Schematic overview of data and sample collection procedures involved in the Celiac Disease Genomic, Environmental, Microbiome, and Metabolomic (CDGEMM) Study.

The CDGEMM study is a multicenter study comprised of collaborators in the United States and Italy. It is supervised by Mass General Hospital for Children at Harvard Medical School in Boston, Massachusetts (Clinical Trials identifier: NCT02061306). CDGEMM aims to study genomic, environmental, microbiome, and metabolomic factors that may contribute to the development of CD longitudinally. In addition to repeated CD serological screening until age five, detailed environmental information is obtained frequently, and stool is collected every three months for the first three years of life and every six months thereafter until age five (Figure 1). Infants’ microbiome and metabolome will be compared longitudinally paying particular attention to differences before and after the introduction of gluten, before and after the development of CD when applicable, as well as many other environmental factors. Additionally, within the longitudinal study we will perform a nested case control analysis. Infants that go on to develop CD with be matched with control infants with a genetic predisposition to, but who have not developed, CD. A second analysis will match infants who go on to develop CD with control infants who do not carry the HLA predisposing genes to address environmental factors that may contribute to alterations in the microbiome and predispose to the development of CD.

CDGEMM was designed with the intent to minimize study visits and maximize participant retention. Innovative data collection techniques allow for remote study recruitment across the United States and other countries. For ease of completion, all questionnaires are distributed through email. Those without access to email or who prefer not to use online data collection techniques may elect to receive their materials by mail and complete all questionnaires on paper. Stool collection materials are sent to the participant for collection, and returned via overnight delivery to our research center where they are immediately frozen at −80 °C [13]. Blood may be collected at a study site or remotely through the same phlebotomy facility that the child’s pediatrician uses. This allows for limitation to a single blood draw at several time points during which sample collection for the study and routine pediatric care overlap. All samples collected remotely are shipped to our research facility overnight, to maintain sample viability, where they are ultimately stored at −80 °C for processing.

### 2.1. Participants

CDGEMM aims to enroll 500 infants aged 0–6 months with a first-degree family member with CD. No more than half of the enrollees will be recruited in Italy. The first study samples must be collected prior to the introduction of solid foods, thus children who have been introduced to solid foods are excluded. The diagnosis of CD in the family member is confirmed by the recruiting institution by review of the pathology report obtained during the confirmatory or diagnostic biopsy. Patients seeking enrollment whose family member did not undergo a confirmatory endoscopic procedure with biopsy are still evaluated for inclusion. Those infants whose family member meets diagnostic criteria based on the published guidelines are accepted for inclusion into the study [14].

### 2.2. Data Collection

The timetable of data collection is shown in Table 1. Health status, anthropometrics, nutritional information, household, and environmental information is obtained regularly. Data is collected through questionnaires, which are distributed directly to the parent’s email address according to a pre-programmed schedule designed around the child’s date of birth. When data is collected in this format, all information gathered is entered directly by participants into a central database using the web-based data collection and management application REDCAP. Parents who are unfamiliar with, or do not have regular access to, a computer are able to receive all questionnaires in pen-and-paper format directly to their home address (through regular mail) according to the study schedule.

### 2.3. Parental and Child Questionnaires

Questionnaires pertain to parental and infant nutrition including infant feeding type, timing of gluten exposure and introduction to other foods, and types and frequency of foods ingested. Information on infant and maternal feeding habits is collected using the Infant Feeding Practices Study II (IFPS II) validated questionnaires created by the Food and Drug Administration (FDA) and Centers for Disease Control and Prevention, in collaboration with other federal agencies [15], modified in order to best assess quantity and frequency of gluten containing and other foods. For the Italian cohort, all instruments were translated and altered slightly for colloquial appropriateness by Italian study staff, with supervision of the principal investigator who is a native speaker of both English and Italian. At enrollment and every six months thereafter, questions regarding medical problems, medication use, including antibiotic use, probiotic use, household members, daytime activity, and other environmental factors are addressed. A symptom diary is included starting at six months of age and vaccinations are documented yearly. A monthly food diary and monthly antibiotic diary during the first year after birth and at 15 months of age collects information to ensure that introduction of foods and any use of antibiotics are recorded accurately.

### 2.4. Serological Markers

Serum is collected every six months for the first three years and then yearly for the remainder of the study (Table 1). An initial sample is obtained prior to the introduction of any solid food for baseline studies. It is analyzed for Immunoglobulin A (IgA) tissue transglutaminase (tTG), using QUANTA Lite Rh-tTG IgA ELISA (INOVA Diagnostics, San Diego, CA, USA). Serology for IgA and Immunoglobulin G (IgG) antideamidated gliadin antibody (dGP) using QUANTA Lite Celiac DGP Screen (INOVA Diagnostics, San Diego, CA, USA) will also be performed. If a patient tests positive for IgG dGP, an IgA level will be determined to investigate the possibility of IgA deficiency. Parents are informed of their child’s serological status throughout the study period—specifically, after each blood draw.

**Table 1:** Detailed schedule of data and samples collected throughout the CDGEMM Study.

Age in Months	0	1	2	3	4	5	6	7	8	9	10	11	12	15	18	21	24	27	30	33	36	42	48	54	60
Maternal Stool and Breast Milk Sample	X																								
Cord Blood	X																								
Blood Sample							X						X *		X		X		X		X		X		X
Stool Sample	X			X			X			X			X	X	X	X	X	X	X	X	X	X	X	X	X
Food Diary		X	X	X	X	X	X	X	X	X	X	X	X	X	X		X		X		X	X	X	X	X
Antibiotic Diary		X	X	X	X	X	X	X	X	X	X	X	X	X											
Maternal Diet	X	X	X	X	X	X	X	X	X	X			X				X				X		X		X
Anthropometrics							X						X		X		X		X		X	X	X	X	X
Medical History	X						X						X		X		X		X		X	X	X	X	X
Parent and Child Demographics	X												X				X				X		X		X
Assessment of Sleep and Activity							X						X		X		X		X		X	X	X	X	X

* HLA testing performed.

### 2.5. Whole Blood

For infants enrolled during gestation, whole blood is collected at birth via cord blood for storage in our biorepository and epigenetic studies. An additional tube of whole blood is collected at one year of age for HLA testing using the DQ-CD screening kit (Biodiagene, Palermo, Italy). Results of the child’s HLA testing are conveyed to parents when the child reaches 18 months of age. DNA is isolated from the remaining blood volume (QIAmp DNA Blood Maxi Kit, QIAGEN, Hilden, Germany) and stored at −20 °C for future use. Excess clotted blood obtained every six months is also stored at −80 °C for future use.

### 2.6. Stool

Stool is collected from the infant at enrollment, which may be as early as seven days after birth. (Figure 1). For children enrolled later in life, enrollment stool samples may be collected anywhere after 15 days and before three months of age. Stool is then collected every three months for the first three years of life and finally every six months thereafter until completion of the study when the child is aged five years. Each stool specimen is collected—from the diaper or from a provided collection hat, depending on the child’s age—into three cryo vials. Two cryo vials contain RNA later solution (Fischer Scientific, NY, USA) to preserve DNA and RNA. A third empty cryo vial will be used for future metabolomic studies. Stool is frozen for 24 h in the participant’s home to ensure sample integrity during shipment and then packaged with a frozen ice pack for overnight delivery to the coordinating research facility [13,16]. Upon arrival to the research facility, all three cryo vials are immediately stored at −80 °C for microbiome and metabolomic studies.

### 2.7. Maternal Samples

Parents who enroll their child during gestation have the option to provide maternal stool and breast milk samples to coincide with the child’s enrollment samples at seven days of age. Like the child’s stool, maternal stool is voided into the stool collection hat and collected into three cryo vials, two of which contain RNA later solution. A small amount of breast milk is also collected concurrently for storage in our biorepository at −80 °C.

### 2.8. Diagnosis of Celiac Disease

Patients testing positive for IgA tTG or IgA dGP or those with IgA deficiency who test positive for IgG dGP will undergo a repeat serological test three months after the initial positive test. If the second serological test returns positive, an anti-endomysial antibody (EMA) will be performed for confirmation. Following two positive serological tests, the patient will be referred to the participating institution, or a pediatric gastroenterologist nearby (if participating remotely), for confirmatory biopsy in order to make a diagnosis of CD.

## 3. Factors of Interest

### 3.1. Environmental

Historically, birthing delivery mode, method of infant feeding and introduction of gluten to the infant diet have been examined for their contributions to the development of CD. Though vaginal delivery and increased duration of maternal breast feeding were once thought to be protective against the development of CD, to date evidence is inconsistent. In terms of birthing delivery mode, the previous association suggested between cesarean delivery and increased incidence of CD [7] seems to no longer be supported [8]. Emilsson and colleagues extracted data from the Norwegian Mother and Child Cohort Study (MoBa), which included approximately 114,000 individuals, to investigate this association and concluded that birth by cesarean section was not associated with increased risk for CD [17]. A recent meta-analysis by Szajewska and colleagues considered results of 21 studies, among which large-scale interventional and observational cohorts were included [9]. This meta-analysis revealed that breastfeeding, whether exclusive or in combination with formula feeding, did not reduce the risk of developing CD. As a collective, the same meta-analysis concluded, in agreement with the Lionetti *et al*., and Vriezinga *et al*., prospective cohort studies [5,6], that timing of introduction of gluten to the infant diet did not significantly influence the risk of developing CD by age three or five years [9]. This is in contrast to the Swedish epidemic of CD presented in 2000, in which a sharp increase in the number of CD cases was noted to be temporally associated with revision of the Swedish national guidelines recommending the introduction of gluten to the infant diet after six months of age [18]. At that time, incidence of CD (per 100,000 births) at two years of age increased from 1.7 to 3.7 cases.

Antibiotic exposure in early infancy has also emerged as a contributory factor to development of CD. Canova *et al.*, found that infections in the first year of life were significantly associated with histologically confirmed CD [10]. A dose dependent effect of antibiotic use has also been reported previously, with increased use of antibiotics serving to increase the risk of developing CD [11]. Socioeconomic status has also been shown to influence development of CD, with CD more frequently observed in children from high SES as compared to low [10]. In fact, rates of autoimmune disease in general were found to be lower in children from lower SES, providing support for the longstanding but controversial hygiene hypothesis [10].

The CDGEMM Study has been carefully designed to address the aforementioned variables, and more, frequently through prospective parent report from the child’s time of birth. Birthing delivery mode is reported by the parent during completion of study enrollment forms. A diary of antibiotic usage is completed by the parents monthly for the first year of life, and food diaries completed at the same time points assess duration of breastfeeding (or other preferred feeding mode) and timing of gluten introduction. After the first year of life, detailed, though less frequent, records of antibiotic use are obtained with each stool sample for the remaining duration of the study. Though the food diary is discontinued at this time, pertinent dietary habits of both the mother and child continue to be carefully recorded approximately every six months.

### 3.2. Genetic

We hypothesize that studies examining environmental factors to date have been inconsistent largely due to the fact that environmental factors contribute to disease development differently based on underlying host factors. While many of the above mentioned environmental factors are likely to be important to the development of CD, it may be the number or the combination of environmental “hits” in the setting of a particular genetic compatibility which influences the microbiome and resulting metabolome to ultimately alter host physiology and lead to the loss of tolerance and development of autoimmune disease. Genetic compatibility with HLA DQ2 and or HLA DQ8 are necessary for the development of CD. Interestingly, the particular genetic makeup has recently been shown to be one of the strongest links to the disease to date. A large-scale study by Lionetti and colleagues recently found that CD developed more frequently in children homozygous for a variant of HLA DQ2 (DQA1*05-DQB1*02) compared to those carrying it only heterozygously [5], confirming previous studies that suggest homozygosity for this allele to be a “high risk” genotype for development of CD [11]. The findings by Lionetti and colleagues are particularly compelling as they suggest a diagnostic utility of early HLA typing of infants at risk for CD and suggest that early environmental factors may contribute to the development of disease differently based on genotype.

### 3.3. Microbiome/Metabolome

It is difficult to isolate the effects of specific environmental variables on the development of CD without taking into account the interplay with the microbiome. The previously reported protective effect of vaginal delivery from developing CD is likely attributable to the variability in microbiome composition that results from different birthing delivery modes [19]. Similarly, antibiotic use early in life has also been shown to relate to increased risk of developing CD [20,21] and could be explained through resulting perturbations to the developing microbiome that come with antibiotic exposure [20]. The CDGEMM cohort provides a unique opportunity to combine environmental data with thorough microbiome, metagenomic, metabolomic, and genetic analytic techniques to investigate these environmental factors and create novel integrative networks to develop a personalized causal predictive model to prevent CD in genetically predisposed individuals.

## 4. Statistical Approach

### 4.1. Power Analysis

**Figure 2:**
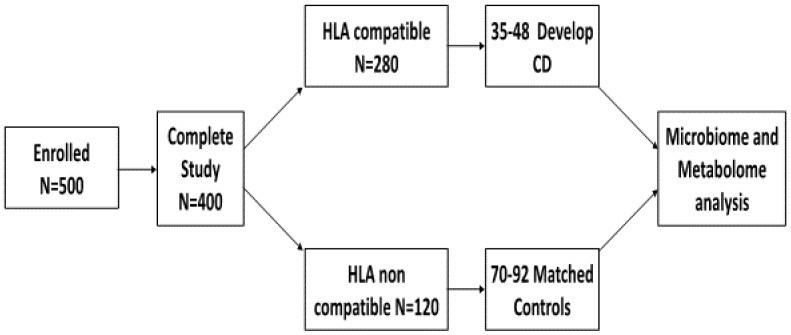
Study scheme outlining the recruitment and projected incidence of celiac disease (CD) and related HLA genotypes for participants in the Celiac Disease Genomic, Environmental, Microbiome, and Metabolomic Study.

Our power assumptions (Figure 2) are based on our recently published data [5,6]. Assuming that: (a) 500 infants will be enrolled during the pilot period and that the drop rate will be no more than 20% given the duration of the study and similar to that we had with our previous studies, [5,6] 400 infants will complete the pilot protocol; (b) the expected probability of CD serum auto antibodies positivity at 18–24 months in first-degree relatives is 10% irrespective of the HLA compatibility; (c) 70% of first degree relatives are HLA DQ2 and/or DQ8 positive (based on our preliminary data); (d) based on points a and b, the expected probability of CD serum auto antibody positivity at 18–24 months in HLA-compatible first degree relatives is 13.3%; (e) the minimal detectable change in CD autoimmunity probability is 10%, we anticipate that during the study period approximately 50 infants will develop CD.

CD is unique among gut inflammatory diseases in that the environmental factor triggering the inflammatory enteropathy is known (gluten) and, therefore traceable in terms of timing of exposure. Given its strong association with HLA DQ2/DQ8, this study provides the ideal control groups, both HLA DQ2/DQ8 negative subjects for subtraction analysis as well as genetically susceptible subjects who have not lost tolerance, to identify metabolites associated with loss of gluten tolerance. Additionally, infants that go on to develop CD will undergo longitudinal analysis, at multiple timepoints, before and after the development of CD.

### 4.2. Developing an Integrative Multilevel Model to Predict Celiac Disease

The CDGEMM Study, with all of its included measures, will serve to create a large database inclusive of environmental and multi-omic information. Thus it will require extensive management and sophisticated computational statistical analysis. We intend to apply integrated data analysis (IDA) which will combine biologic, genetic, and environmental data from participants in CDGEMM. Integration of raw multi-omic data to create predictive models for the development of CD will be a crucial innovation in order to advance knowledge about this multi-factorial disease.

Multilevel mediation-moderation models allow for modeling the correlation of clustered data (e.g., time points within individuals) and to analyze the effect of a factor as a possible bridge (mediator) or attenuator/amplifier (moderator) of a relationship between two other variables. These mediation-moderation models will be used with data from infant and parent samples, integrating serologic data, genetic data, and environmental factors—including mode of infant delivery, dietary regimen, antibiotic intake, and many others—with microbial information and metabolic pathway phenotypes to elaborate causation models that can detect specific individual patterns leading to the loss of tolerance to gluten.

Specifically, we will examine (1) the genomics effects on proteomics mediated by the microbiome and metabolic activity on the final proteome; (2) the effect of the environment (region, rural *versus* urban) on the intestinal microbiome and the metabolites produced; and (3) the mediation effect of metatranscriptomics on the onset of CD. Further description and identification of the mechanism by which environmental and lifestyle factors (e.g., diet, antibiotic intake) contribute to the loss of tolerance to gluten and development of CD are desperately needed. Factors involved are likely to vary on individual aspects such as HLA genotype; timing, frequency, and type of antibiotic intake; dietary regimen; and birthing delivery mode, but studies to date have not examined these factors in combination. The proposed research aims to test potential mechanisms by examining the interaction among a wide range of factors. Our findings may help to establish personalized strategies to address the etiology of losing tolerance to gluten through the identification of predictive biomarkers thus allowing us to predict CD. The model will additionally be applicable to other autoimmune diseases such as type-1 diabetes mellitus.

**Figure 3:**
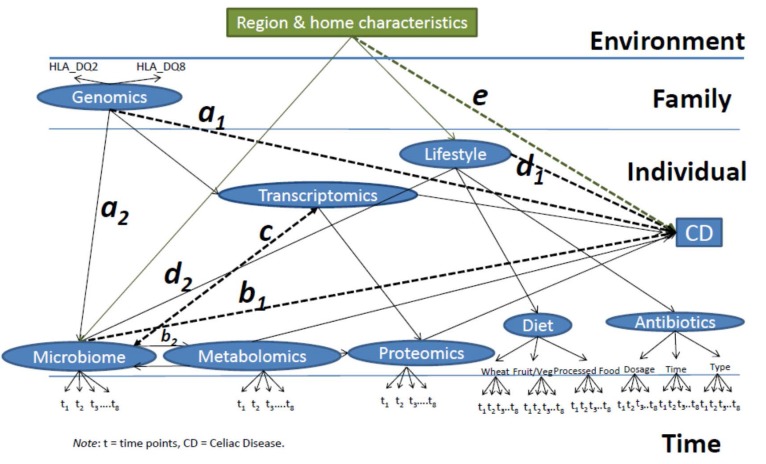
Celiac Disease Genomic, Environmental, Microbiome, and Metabolomic (CDGEMM) Integrative Multilevel Model to Predict Celiac Disease.

The conceptual framework proposed (Figure 3) draws on biological and environmental literature [22,23,24,25] examining the intersection between biomarkers, environmental factors, genetic factors, and individual factors. The onset of loss of tolerance to gluten will be measured as a continuous dependent variable based on levels of tTG IgA. Ultimately the diagnosis of CD will be confirmed by an endoscopy with duodenal biopsy. Given this, we expect to observe the following:

(1) *Path a*: a causal path between the *genetics* and the final effect on the *onset of CD*, with the *microbiome* as the mediator (a_1_ × a_2_), such that the intestinal microbiome will be altered in order for the genetics to ultimately influence the development of disease (microbiome-mediated epigenetic pressure);
(2) *Path b*: a causal path of the *microbiome’s* effect on *CD* mediated (b_1_ × b_2_) by the *metabolic* activity of intestinal bacteria, which, through the production of metabolites, affects the intestinal *transcriptome* and *proteome*. Including a bidirectional path between microbiome and metabolomics to capture how metabolism could alter the microbial metabolism and ultimately the risk for CD;
(3) *Path c*: A bidirectional path between the *transcriptome* and the *microbiome* as their causal relationship will depend on each other;
(4) *Path d*: a direct relationship between *lifestyle factors* (here represented by dietary regimen and antibiotic intake) and the onset of the disease mediated (d_1_ × d_2_) by alterations in the *microbiome*;
(5) *Path e*: The *environment* (including region characteristics, urban compared to rural, number of individuals living in the household, birth order, pets present) affecting the *onset of the disease* as a mediator and affecting the composition of the *microbiome* and resulting *metabolome*, in turn, influencing gene and protein expression;
(6) *Full Model*: Time points will be clustered within individual characteristics, analyzing all the paths listed above (Figure 3), individuals will be clustered within families, and families within regions.

## 5. Conclusions

The implementation of primary prevention strategies for CD through manipulation of the microbiota would represent a complete shift of paradigm in autoimmune pathogenesis and treatment of life-long autoimmune disorders. The identification of specific CD metabolomic phenotypes can also help to define additional diagnostic tools and therapeutic interventions. Additionally, CDGEMM’s biorepository will allow for future epigenetic studies and validation of biomarkers. Our findings may have a far-reaching impact on other pediatric autoimmune diseases in which the diet-genome-microbiome interaction in the pathogenesis of the disease has been hypothesized. This, in turn, could help to set up strategies aimed at readdressing the process of oral tolerance to gluten and to other environmental antigens, opening the way to novel approaches of prevention/treatment of autoimmune diseases. Since 3 million people in the U.S. are affected by CD and approximately 17 million people suffer from other autoimmune diseases, and there currently are no effective strategies to prevent these conditions, the findings from CDGEMM can potentially have a tremendous impact on pediatric public health [26].
